# Patient-Reported Outcomes from a Pilot Plant-Based Lifestyle Medicine Program in a Safety-Net Setting

**DOI:** 10.3390/nu15132857

**Published:** 2023-06-24

**Authors:** Rachel E. Massar, Michelle McMacken, Lorraine Kwok, Shivam Joshi, Sapana Shah, Rebecca Boas, Robin Ortiz, Lilian Correa, Krisann Polito-Moller, Stephanie L. Albert

**Affiliations:** 1 Department of Population Health, New York University Grossman School of Medicine, New York, NY 10016, USA; lorraine.kwok@nyulangone.org (L.K.); stephanie.albert@nyulangone.org (S.L.A.); 2 Department of Medicine, New York University Grossman School of Medicine, New York, NY 10016, USA; michelle.mcmacken@nyulangone.org (M.M.); shivam.joshi@nyulangone.org (S.J.); sapana.shah@nyulangone.org (S.S.); rebecca.boas@nyulangone.org (R.B.); 3 Department of Medicine, NYC Health + Hospitals/Bellevue, New York, NY 10016, USA; lilian.correa@nychhc.org (L.C.); politok@nychhc.org (K.P.-M.); 4 Office of Ambulatory Care and Population Health, NYC Health + Hospitals, New York, NY 10004, USA; 5 Division of Nephrology, Department of Medicine, Veterans Affairs, Orlando, FL 32827, USA; 6 Departments of Pediatrics and Population Health, Institute for Excellence in Health Equity, NYU Langone Health, New York, NY 10016, USA; robin.ortiz@nyulangone.org

**Keywords:** plant-based diet, lifestyle medicine, behavior change, lifestyle modification, lifestyle intervention

## Abstract

Lifestyle medicine interventions that emphasize healthy behavior changes are growing in popularity in U.S. health systems. Safety-net healthcare settings that serve low-income and uninsured populations most at risk for lifestyle-related disease are ideal venues for lifestyle medicine interventions. Patient-reported outcomes are important indicators of the efficacy of lifestyle medicine interventions. Past research on patient-reported outcomes of lifestyle medicine interventions has occurred outside of traditional healthcare care settings. In this study, we aimed to assess patient-reported outcomes on nutrition knowledge, barriers to adopting a plant-based diet, food and beverage consumption, lifestyle behaviors, self-rated health, and quality-of-life of participants in a pilot plant-based lifestyle medicine program in an urban safety-net healthcare system. We surveyed participants at three time points (baseline, 3 months, 6 months) to measure change over time. After 6 months of participation in the program, nutrition knowledge increased by 7.2 percentage points, participants reported an average of 2.4 fewer barriers to adopting a plant-based diet, the score on a modified healthful plant-based diet index increased by 5.3 points, physical activity increased by 0.7 days per week while hours of media consumption declined by 0.7 h per day, and the percentage of participants who reported that their quality of sleep was “good” or “very good” increased by 12.2 percentage points. Our findings demonstrate that a lifestyle medicine intervention in a safety-net healthcare setting can achieve significant improvements in patient-reported outcomes. Key lessons for other lifestyle medicine interventions include using a multidisciplinary team; addressing all pillars of lifestyle medicine; and the ability for patients to improve knowledge, barriers, skills, and behaviors with adequate support.

## 1. Introduction

Lifestyle behaviors such as diet, physical activity, sleep, substance use and social relationships are key modifiable risk factors in the development of chronic diseases [1,2,3]. As a medical specialty, lifestyle medicine uses therapeutic lifestyle interventions that focus on improving lifestyle behaviors as a primary method to treat chronic conditions [4]. Lifestyle medicine is becoming increasingly popular in the U.S. due to growing recognition of its benefits, particularly in terms of cardiometabolic health [4,5,6,7,8,9,10,11,12]. Such interventions consist of evidence-based care delivered by trained clinicians with a focus on the pillars of lifestyle medicine, which include a healthful plant-predominant eating pattern, regular physical activity, restorative sleep, stress management, positive relationships, and avoidance of risky substances. Training and board certification in lifestyle medicine are available for healthcare practitioners to support the promotion of high-quality, evidence-based lifestyle interventions. Lifestyle medicine has the potential to significantly improve many chronic conditions, such as coronary heart disease, type 2 diabetes, hypertension, hyperlipidemia, and nonalcoholic fatty liver disease; in some cases, it can even induce remission of type 2 diabetes [13,14]. For this reason, guidelines from major medical societies emphasize lifestyle modification as first-line therapy, or a critical component of first-line therapy, for cardiovascular disease, type 2 diabetes, obesity, and cancer risk reduction [15,16,17,18,19,20,21].

Historically, formal lifestyle medicine programs have primarily been offered as part of employee health, pay-out-of-pocket, or residential programs rather than in traditional healthcare settings. However, lifestyle medicine is now being offered within healthcare systems, as evidenced by the growth of the Health Systems Council of the American College of Lifestyle Medicine [22]. More than 80 major health systems in the U.S. have joined the Council, signaling their commitment to integrating lifestyle medicine into their patient services. The increasing growth of lifestyle medicine within health systems is likely driven by a combination of factors, including patient demand, interest in value-based care, concern for rising healthcare costs, and recognition that health behavior change is fundamental to the burden of chronic disease and its complications [4].

As lifestyle medicine proliferates, it is critical that these interventions are accessible to a wide variety of populations, especially Hispanic and Black individuals, who face the highest burden of chronic disease including diabetes and hypertension [23]. Black individuals and those who are low-income also have a higher prevalence of multimorbidity [24]. Safety-net healthcare settings play a significant role in providing medical care to individuals who are low-income and are either uninsured or receive Medicaid benefits [25]. In 2021, safety-net health centers served more than 30 million patients, including one in three people living in poverty in the U.S. [26]. The patient population served by safety-net health settings also bears a disproportionate burden of adverse health outcomes, including lifestyle-related chronic diseases [23]. Integrating lifestyle medicine into safety-net healthcare settings represents an important opportunity to treat those who face a high risk of lifestyle-related chronic illness as well as significant structural barriers to making lifestyle changes in the first place. Arguably, these individuals stand to benefit the most from lifestyle medicine interventions along with specific measures to address social needs.

Given the strong emphasis on positive behavior change in lifestyle medicine, evaluations of lifestyle medicine programs should prioritize the assessment of proximal patient-reported outcomes such as knowledge and barriers to change, as well as patient-reported lifestyle behaviors themselves. In addition, the evaluation of program efficacy by patient-centered outcomes such as self-rated health and quality of life, instead of a narrow reliance solely on clinical outcomes, can provide important insights into the impact of lifestyle medicine on patients’ lives. Previous studies of lifestyle medicine interventions have demonstrated improvements in self-reported dietary behaviors [12,27,28,29,30], physical activity [12,30,31], self-rated health/well-being [32,33,34], and quality-of-life [35]; however, all of these interventions were set outside the traditional healthcare setting in worksite, community-based, or research settings while no studies to date have studied the impact of a lifestyle medicine program implemented in a safety-net setting. 

This study builds on previously reported implementation and clinical outcome findings and aims to assess patient-reported outcomes on nutrition knowledge, barriers to adopting a plant-based diet, food and beverage consumption, lifestyle behaviors, self-rated health, and quality-of-life for participants in the pilot plant-based lifestyle medicine (PBLM) program. The pilot PBLM program is a lifestyle medicine intervention set within a safety-net healthcare setting in New York City with the goal of reducing cardiometabolic risk in patients through healthy lifestyle behavior changes. To our knowledge, this is the first study to publish patient-reported outcomes from a lifestyle medicine intervention set within a traditional safety-net healthcare setting. Given the recent growth of lifestyle medicine in U.S. health systems, the findings of this study provide key practice implications for healthcare practitioners seeking to implement similar interventions within traditional healthcare settings, and particularly in safety-net settings.

## 2. Methods

All study procedures were approved by the New York University Grossman School of Medicine (NYU) IRB (s18-01319) as well as the Office of Research and Administration for Implementation at NYC Health + Hospitals/Bellevue. 

### 2.1. Intervention

The PBLM program was a one-year pilot clinical program implemented in an adult primary care setting in a large urban safety-net healthcare system in New York City. Details of and updates made to the PBLM program have been described in detail elsewhere [36]. In brief, the program was designed to help patients reduce their cardiometabolic risk through positive lifestyle changes. Adults with type 2 diabetes, prediabetes, heart disease, high blood pressure, high cholesterol, and/or excess weight (BMI ≥ 25) were eligible to participate. The goals of the program were to encourage patients to transition to a healthful plant-based eating pattern, improve sleep, increase physical activity, improve stress management, increase social connection, and avoid substance use. The provider team comprised four physicians, one registered dietitian, and one health coach. Each physician has expertise in plant-based nutrition, as well as their own specialties in medicine including internal medicine, cardiology, nephrology, and muscular–skeletal disorders. The registered dietitian is trained in community-focused nutrition and is also a trained chef. The health coach has certifications in plant-based nutrition, yoga therapy, and personal fitness training. Participants met individually with providers to set goals and monitor progress. Group classes supplemented one-on-one visits, emphasizing education, skills building, and peer-to-peer support. In addition, an exercise trainer offered classes focused on aerobic and strength training. Resources available to all participants included a plant-based diet starter guide written by program providers, plant-based cookbook(s), coupons that could be used to purchase fresh fruits and vegetables at all NYC farmer’s markets, a Healthy Savings Card for grocery store discounts on fresh produce, and access to a private Facebook group for social support, resource ideas, and recipe sharing. The frequency and length of program engagement were jointly determined by the PBLM provider team and each of the participants. This description of the PBLM program reflects its design at the outset of the pilot phase. As anticipated, continuous assessments and adjustments were made throughout the pilot period to adapt to the realities of working within a complex healthcare setting, as well as to meet the evolving needs of the participants. Significant improvements in clinical cardiometabolic outcomes, including reductions in weight, hemoglobin A1c, and diastolic blood pressure, have been previously reported from this pilot program [37].

### 2.2. Sample 

This study makes use of patient-reported data collected as part of the evaluation of the pilot PBLM program. However, the primary focus of the evaluation was to assess the feasibility of implementation and demand for the program. Recruitment for the evaluation happened in two phases. First, the coordinating manager told all individuals who scheduled an initial appointment with the PBLM program about the study and asked for permission to share their contact information with the NYU evaluation team (*n* = 173). The NYU evaluation team then reached out to only those individuals who agreed to be contacted (*n* = 131) to provide additional information and obtain verbal consent. One hundred and eleven individuals (85%) agreed to participate in the evaluation. Subsequently, 109 individuals completed a baseline survey (98%), 93 individuals completed a 3-month survey (84%), and 84 individuals completed a 6-month survey (76%). Baseline surveying began on 29 January 2019 and continued until 30 July 2019. The 3-month assessment occurred between 18 April 2019 and 25 November 2019 and the 6-month data were collected between 30 July 2019 and 26 February 2020. Surveys were administered telephonically by trained evaluation staff and took approximately 25 min to complete. Respondents were given a USD 10 gift card each time a survey was completed to thank them for their time and effort. The current study utilizes a subsample of survey completers. Participants were included in this study only if they completed the survey at all three time points and if they confirmed any level of participation in the PBLM program. Some individuals who initially registered for the program did not actually join due to various factors including schedule conflicts or insurance issues. Therefore, 71 individuals were eligible to be part of the sample for the present study. 

### 2.3. Survey Measures

In the current study, five outcome domains were assessed among participants: (1) nutrition knowledge, (2) barriers to adopting a plant-based diet, (3) food and beverage consumption, (4) lifestyle behaviors, and (5) self-rated health and quality of life. 

#### 2.3.1. Nutrition Knowledge 

In order to assess knowledge about plant-based diets and nutrition, respondents were asked to indicate whether each of eight statements was true or false. Questions were developed de novo by the research team and included items such as “*It’s not healthy to eat a lot of carbohydrates*” and “*Diets without meat are too low in protein*”. These knowledge questions had only one correct response and were coded as “correct” or “incorrect”. For analyses, a variable was calculated for the percent correct across the eight knowledge questions.

#### 2.3.2. Barriers to Adopting a Plant-Based Diet

To assess real or perceived barriers to adopting a plant-based diet, we asked participants to indicate to what extent they agreed or disagreed with 21 different statements [38]. Examples of statements posed to respondents include “*You are not sure how to be healthy on a plant-based diet*” and “*You don’t want to change your eating habits or routine*”. For analyses, we dichotomized responses to “strongly disagree/disagree” vs. “agree/strongly agree”. We then constructed a variable to represent the number of barriers endorsed by a person. The range of plausible values was between 0 and 21. 

#### 2.3.3. Food and Beverage Consumption

Food and beverage consumption was measured using 16 questions about food and beverages such as “In the last 7 days, how often did you eat vegetables such as lettuce, spinach, broccoli, cauliflower, carrots, peas, corn, tomatoes?” and “In the last 7 days, how often did you eat beef, pork or lamb?”. Items were adapted from the PrimeScreen dietary screening tool [39]. Additionally, the research team developed items de novo. Responses to all items ranged from “No times during the past 7 days” to “3 or more times per day”. For analyses, each question was treated as a pseudo-continuous variable and was constructed such that it represented the average number of times a food or beverage was consumed per week. Responses were coded as follows: no times at all = 0, 1–3 times during the past 7 days = 2, 4–6 times during the past 7 days = 5, 1 time per day = 7, 2 times per day = 14, 3 or more times per day = 21. 

To calculate a composite food and beverage consumption score, we created a modified Healthful Plant Based-Diet Index (hPDI) [5]. We determined that it was necessary to modify the original hPDI because our survey only collected information on a limited number of food and beverage items, the time frame was shorter, and the quantification of consumption was measured differently in our study. Similar to the original hPDI, we categorized food and beverage consumption items into three groups: healthier plant-based foods, less healthy plant-based foods, and animal foods. Healthier plant-based foods included: whole-grain foods, fruits, vegetables (including potatoes but excluding fried potatoes/chips), nuts or seeds, and legumes, tofu or tempeh. Plant-based meats and seitan were excluded, as some forms of these foods may be considered healthy and others less healthy; the survey instrument did not allow us to categorize. Less healthy plant-based foods included: refined grain foods, sugar-sweetened beverages (i.e., regular soda, punch, sports drinks, energy drinks, or sweetened fruit juice), and sweets. Animal foods included: dairy, eggs, fish or seafood, and meat (including processed meat, beef, pork, lamb, or chicken). Healthier plant-based foods were coded as follows: no time at all = 0, 1–3 times during the past 7 days = 1, 4–6 times during the past 7 days = 2, 1 time per day = 3, 2 times per day = 4, 3 or more times per day = 5. Less healthy plant-based foods and animal foods were reverse-coded. The food group scores were then summed to create a modified hPDI score. Higher scores indicated a healthier overall dietary pattern characterized by a higher intake of healthy plant-based foods and lower intake of less-healthy plant-based and animal foods. 

#### 2.3.4. Lifestyle Behaviors

To measure lifestyle behaviors, we asked respondents to report on their physical activity, media consumption, and sleep hygiene. Respondents were asked: “*In the last 7 days, how many days did you exercise for 30 min or more at a moderate to strenuous intensity in your free time? Moderate to strenuous intensity would be brisk walking or enough movement to break a light sweat*”. This item was adapted from the Lifestyle Assessment Long Form, Physician Version [40]. We also included a measure of media consumption as a proxy for sedentary behavior. Specifically, respondents were asked: “*On a typical day, how many hours do you spend using media for things other than work? Include the time you spend watching TV, videos or movies, gaming, using social media, or visiting websites*” [41]. Viable response options ranged from 0 to 24. Lastly, three questions measured sleep duration and quality. These items were adapted from the Pittsburgh Sleep Quality Index [42]. Specifically, respondents were asked: “*During the past month, how many hours of sleep do you get on a week night, that is Sunday-Thursday*?”. This question was repeated to measure weekend sleep. To measure sleep quality, we asked “*During the past month, how would you rate your sleep quality overall?*” using a 4-point Likert scale from were “very good” to “bad”. Response options were dichotomized to “very good/fairly good” versus “fairly bad/bad”.

#### 2.3.5. Self-Rated Health and Quality-of-Life

Respondents were asked to rate their health on a scale from 0 (very poor health) to 10 (excellent health) [40]. Participants were also asked to rate their quality of life over the past month using a 5-point Likert scale from “very poor” to “very good” [43]. Response options were collapsed to “good or very good” versus “fair, poor, very poor”.

Demographic measures collected included gender, age (in years), race/ethnicity, language use, education (less than high school, high school, some college, college/more than college), health insurance (private, public, no insurance, other), marital status (single, married/living with a partner, separated/divorced, widowed), number of household residents, and food security (often we don’t have enough to eat, sometimes we don’t have enough to eat, we have enough to eat but not always the kinds of good we want, we always have enough to eat and the kinds of food we want).

### 2.4. Statistical Analyses

We assessed changes in the study outcomes between the three survey administrations. For each outcome, we assessed changes from baseline to the 3-month survey, changes between 3 and 6 months, and changes from baseline to the 6-month follow-up. These tests were intended to assess immediate/short-term changes in patient-reported outcomes (baseline to 3 months), retention of changes (3 to 6 months), and longer-term changes (baseline to 6 months). We performed paired *t*-tests for continuous variables and McNemar’s tests for paired dichotomous variables. All analyses were conducted using Stata/SE version 14.2 [44]. Results were considered statistically significant if they had a *p*-value less than 0.05. 

## 3. Results

Table 1 shows the demographic characteristics of the PBLM program sample. The majority of the sample was female (67.6%), participants were on average 55 years old, a little over one-third of participants (38.6%) identified as White and 31.4% identified as Black/African American, and the majority spoke only English at home (85.9%). The majority of participants reported their highest educational attainment was college or more than college (67.6%), had private insurance (57.1%), and were not experiencing food insecurity (98.5%). Lastly, the sample mostly comprised individuals who reported being single or married/living with a partner (43.7% and 40.8%, respectively), and on average, people lived in a household with two residents.

### Survey Findings

Table 2 displays changes in participants’ nutrition knowledge, the number of barriers to adopting a plant-based diet, food and beverage consumption, lifestyle behaviors, self-rated health, and quality of life over time.

Overall, there was a statistically significant increase in participants’ nutrition knowledge from baseline to 3-month follow-up (66.4% vs. 71.5%; *p* = 0.005), and this was maintained at 6 months (66.4% vs. 73.6%; *p* = 0.001). The total number of barriers to adopting a plant-based diet decreased from baseline to 3 months (7.2 vs. 5.7; *p* = 0.002) continued to decrease significantly between 3 and 6 months (5.7 vs. 4.8; *p* = 0.005) and persisted at 6 months (7.2 vs. 4.8; *p* < 0.001). Out of 21 total barriers, 4 decreased significantly from baseline to 3 months: *not being sure how to be healthy on a plant-based diet* (52.9% vs. 29.4%; *p* < 0.001); *not knowing how to prepare plant-based meals* (55.1% vs. 29.0%; *p* < 0.001); *not knowing what to eat on a plant-based diet* (53.6% vs. 17.4%; *p* < 0.001); and *not having read or heard much about a plant-based diet* (25.4% vs. 9.9%; *p* < 0.001) (see Supplementary Table S1). Between 3 and 6 months, one barrier, *not being sure how to be healthy on a plant-based diet*, continued to decrease significantly (29.4% vs. 13.0%; *p* = 0.003). In addition to these four barriers, three additional barriers were statistically different from baseline at 6 months: *not having enough willpower to change to a plant-based diet* (26.1% vs. 12.9%; *p* = 0.031); *craving meat, dairy, or eggs* (46.5% vs. 31.0%; *p* = 0.013); and *not knowing anyone who eats a plant-based diet* (43.7% vs. 28.2%; *p* = 0.035).

Regarding the consumption of healthier plant-based foods, we found a statistically significant increase in the average number of times participants consumed whole-grain foods (5.5 vs. 8.3; *p* < 0.001), fruits (7.7 vs. 11.6; *p* < 0.001), vegetables (9.3 vs. 12.6; *p* < 0.001), as well as legumes, tofu, and tempeh (2.7 vs. 3.8; *p* < 0.001) per week at 3 months. There was a statistically significant decrease in vegetable consumption between 3 and 6 months (12.6 vs. 11.2; *p* = 0.044) but an overall significant increase from baseline to 6 months (9.3 vs. 11.2; *p* = 0.008). Change at 6 months was also significant for the consumption of fruits (7.7 vs. 10.2; *p* = 0.002), and legumes, tofu, and tempeh (2.7 vs. 3.9; *p* < 0.001). The consumption of less healthy plant-based foods decreased over time. Specifically, the consumption of refined grain foods decreased significantly from baseline to 3-month follow-up (3.8 vs. 1.9; *p* < 0.001) and was maintained at 6 months (3.8 vs. 2.0; *p* < 0.001). We also found a significant 6-month reduction in the consumption of sugar-sweetened beverages (0.8 vs. 0.4; *p* = 0.011). Additionally, there was a 3-month decrease in participants’ consumption of sweets (4.0 vs. 2.6; *p* = 0.009) that persisted at 6 months (4.0 vs. 2.3; *p* = 0.002). Participants’ consumption of animal foods (i.e., dairy, eggs, and meat) declined significantly from baseline to 3-month follow-up (dairy: 4.2 vs. 1.5; *p* < 0.001; eggs: 2.4 vs. 1.0; *p* < 0.001; meat: 4.7 vs. 1.9; *p* < 0.001) and was maintained at 6 months (dairy: 4.2 vs. 1.6; *p* < 0.001; eggs: 2.4 vs. 0.8; *p* < 0.001; meat: 4.7 vs. 2.0; *p* < 0.001). We found statistically significant 6-month reductions in consumption of fish and seafood (1.7 vs. 1.0; *p* = 0.021). The modified hPDI score significantly increased from 37.8 to 43.9 (*p* < 0.001) between baseline and 3-month follow-up and was maintained at 6 months (37.8 to 43.1; *p* < 0.001). 

There were some improvements in participants’ lifestyle behaviors such as physical activity, daily media consumption, and sleep quality. At baseline, PBLM participants reported engaging in 30 min or more of moderate to strenuous physical activity about 2 days per week. There was an increase in physical activity between baseline and 3 months (2.3 vs. 2.9; *p* = 0.017) that was sustained at 6-month follow-up (2.3 vs. 3.0; *p* = 0.036). Daily media consumption (watching TV, videos or movies, gaming, using social media, or visiting websites for things other than work) dropped significantly from baseline to 6 months (3.7 vs. 3.0 h per day; *p* = 0.022). At baseline, 55% of participants indicated that they had “fairly good” or “very good” sleep quality. The percentage of participants increased to about 65% at 3 months, but this immediate change was not statistically significant. Improvement in sleep quality was observed (54.9% vs. 67.1% “fairly good” or “very good” sleep quality; *p* = 0.035) at 6 months. 

There was an increase in participants’ average self-rated health score (5.9 vs. 6.6 out of 10; *p* = 0.001) from baseline to 3 months that was sustained at the 6-month follow-up (5.9 vs. 6.5; *p* = 0.038). No statistically significant changes were found in the percentage of people who reported that their quality of life was “good” or “very good” over time.

## 4. Discussion

**Figure 1:**
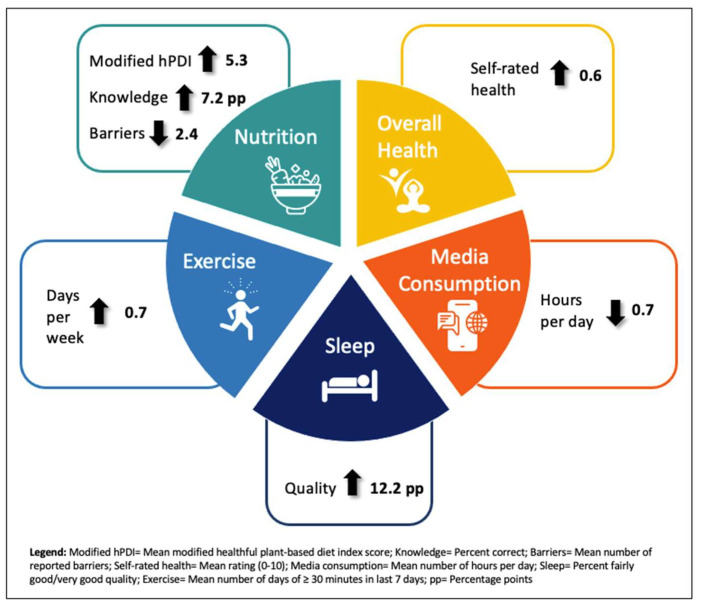
Self-reported improvements among the PBLM program sample at 6 months.

This study aimed to assess patient-reported outcomes related to nutrition knowledge, barriers to eating a plant-based diet, food and beverage consumption, lifestyle behaviors, and overall health and quality-of-life from participants of a pilot plant-based lifestyle medicine program within a traditional safety-net healthcare setting. Our findings show that participants reported statistically significant and immediate improvements at 3 months, maintained improvements for the majority of outcomes between 3 and 6 months, and achieved longer-term improvements, overall, from baseline to 6 months of participation. Specifically, after 6 months, nutrition knowledge increased, participants reported fewer barriers to adopting a plant-based diet, the score on a modified healthful plant-based diet index increased, weekly physical activity increased while daily hours of media consumption declined, and the percentage of participants who reported their quality of sleep was “good” or “very good” increased (see Figure 1). Sleep duration and quality of life were the only outcomes of interest where no change was detected. Our findings are consistent with previous literature that found improvements in self-reported eating behaviors [12,27,28,29,30], physical activity [12,30,31], and self-rated health/well-being [32,33,34] through lifestyle medicine interventions. However, our study is novel because it is the first to show that such improvements can be achieved through participation in a lifestyle medicine intervention within a traditional safety-net healthcare setting. Previous reports have focused on lifestyle programs in worksite, community, and research settings. 

Our findings show that participants gained more nutrition knowledge and reported fewer barriers to eating a plant-based diet after participating in the program. Out of the seven barriers that declined significantly, three had rather sizeable reductions over time in the percent of participants endorsing them as barriers: *not knowing how to prepare plant-based meals* (34.5 percentage points), *not being sure how to be healthy on a plant-based diet* (39.9 percentage points), and *not knowing what to eat on a plant-based diet* (42 percentage points). This finding suggests that while these barriers were common amongst participants at baseline, they were thoroughly addressed through knowledge and skills gained through participation in the PBLM program. Alternatively, there were four barriers that remained resistant to change over time from baseline to 3 and 6 months: *not having enough choice when eating out* (68.6%; 67.1%; 60.0%); *too much planning* (54.0%; 49.2%; 42.9%); *your family or partner won’t eat a plant-based diet* (51.7%; 43.1%; 40.4%), and *you would have to go food shopping too often* (42.9%; 45.7%; 40.0%). Some of these barriers may be outside of the scope of the PBLM program (family and partners’ food choice; eating out), while others may require more attention in future lifestyle medicine interventions in order to overcome them (planning and food shopping). In addition, there were three items that were rarely cited as being barriers to adopting a plant-based diet during the baseline, 3-month, and 6-month survey administrations: *someone else decides on most of the food you eat* (11.3%; 4.2; 8.5%); *you don’ like the taste of many of the foods that are the foundation of a plant-based diet* (8.7%; 10.1%; 7.1%); and *you don’t want to change your eating habits or routine* (7.0%; 7.0%; 7.1%.) These findings suggest that the PBLM participants were autonomous in their food choices, open to eating plant-based foods, and highly motivated to modify their eating behaviors. 

We found a consistent pattern of improvement in reported intake of healthful plant-based foods, as well as improvement in participants’ overall plant-based diet, as measured by the modified hPDI composite score. These results align with the strong emphasis the PBLM program placed on helping participants transition to a healthful plant-based eating pattern. Participants worked with a physician who validated the health benefits of this dietary approach, a registered dietitian who provided nutrition education and medical nutrition therapy tailored to individual needs, and a health coach who provided support in implementing the recommended nutrition plan in participants’ everyday lives. It is worth noting that there was a small, but statistically significant, decrease in consumption of vegetables between the 3-month and 6-month surveys. However, this measure improved significantly overall from baseline to 6 months of program participation. The small dip in the consumption of vegetables may be explained by challenges with long-term adherence to healthy behavior changes. Middleton et al. found that initial responses to lifestyle interventions are often encouraging but are frequently followed by declines in adherence over time [45]. Most salient in our findings, however, is the overall positive pattern of improvement in the consumption of healthy plant-based items individually, as well as the comprehensive healthy plant-based diet improvement from baseline to 6 months. In addition, a previous article documented that PBLM program participants experienced clinical improvements that are closely associated with healthful diet changes including weight loss, and reductions in HbA1c and blood pressure [37], providing further evidence of this study’s overall positive findings for healthy diet improvement. 

In addition to focusing on dietary changes, the PBLM program emphasized increasing physical activity among patients. The health coach advised patients on ways to incorporate more physical activity into their daily lives, and the program offered aerobic and strength training classes using program-supplied resistance bands. Our findings show that patients improved their amount of physical activity over time, which is in line with findings from previous studies evaluating lifestyle medicine programs [12,30,31]. Participants also significantly reduced their media consumption outside of work activities, which could suggest less sedentary behavior following their engagement with the program. 

We found that the percentage of participants who reported good sleep quality improved significantly over time. Through individual sessions with each of the lifestyle medicine team members and group classes with the dietitian and health coach, participants received guidance on enhancing sleep quality and duration. Furthermore, due to the interconnectedness of lifestyle behaviors, improvement in sleep quality may have been influenced by the other healthful lifestyle changes participants made. Diet, physical activity, and sleep are all closely related, and the improvement of one behavior can positively impact another [46,47,48]. Interestingly, while we found that sleep quality improved significantly, we were not able to detect a significant improvement in participants’ sleep duration. Participants’ average reported sleep duration ranged from 6.3 to 6.5 h during weeknights and 6.9 to 7.3 h on weekend nights during the three survey periods, falling slightly below the recommended number of hours of sleep for adults (7–9 h) [49]. Our findings suggest that improving the sleep duration is challenging through lifestyle medicine intervention. Sleep health is multifactorial and highly affected by one’s environment and routine [50]. Modern challenges to sleep health include the negative impact of digital screens and the discrepancy between the body’s circadian rhythm and schedule demands [50]. 

Importantly, the PBLM program was centered on weight-inclusive principles that focused on outcomes related to overall health and well-being [51]. The program team sought to provide non-stigmatizing care to participants by shifting the primary goal from weight loss to emphasizing incremental, achievable, pleasurable, and sustainable healthful behavior changes. Their approach to providing care to individuals with diverse body sizes aimed to reduce the stigma often experienced by patients in clinical settings that has been associated with suboptimal healthcare utilization [52]. This study affirms the weight-inclusive approach by including self-rated health and quality of life as important indicators of programmatic successes. We found that self-rated health improved significantly, but we were not able to detect an appreciable change in the quality of life. Our lack of statistically significant quality-of-life findings may be due to the multidimensional nature of this construct that encompasses much more than just health and health-related indicators. 

Many providers may question their patients’ willingness and ability to make dietary changes [53], especially to a plant-based eating pattern [54]. Our findings support the feasibility of a plant-based dietary approach as part of a team-based lifestyle medicine intervention. With education and guidance from the clinical team, patients in this program reported significantly fewer barriers to adopting a plant-based diet, as well as significant movement towards a healthful plant-based eating pattern. The program’s private Facebook page and group classes were designed to promote peer support and facilitate long-term dietary behavior change. The persistence of participants’ reported dietary changes over time suggests that participants did not view this approach as a temporary “diet”, but rather as a lifestyle change that is meant to be sustainable. 

Although the program placed primary emphasis on nutrition, in many cases other pillars of lifestyle change were upstream of patients’ ability to make dietary changes. Poor-quality and/or insufficient sleep, for example, emerged anecdotally as a key barrier to patients’ ability to change their eating patterns. “Emotional eating” and other challenges in coping with stress also greatly affected participants’ dietary behaviors. As the program evolved, the need to place additional focus on the non-nutrition pillars of lifestyle medicine became evident. A comprehensive approach, addressing all aspects of lifestyle medicine, is likely foundational to participants’ capacity to optimize health behaviors.

Our study has limitations that should be acknowledged. First, this study relied on self-reported behaviors, including a brief food screener, that are subject to recall and reporting biases. While food screeners are a common and relatively easy way of measuring food and beverage consumption, retrospective questions such as these are cognitively burdensome and have been found to result in biased estimates [55]. Second, there may be measurement error associated with our food and beverage screener. The maximum number of times a respondent was able to report consuming a food or beverage was three times per day; therefore, potentially underestimating the number of times a food category was consumed. Third, this study did not measure important pillars of lifestyle medicine including positive social relationships and stress management that future research should address. Fourth, this study employed a single sample pre-repeated post-test design. As such, there was no comparison group and causality cannot be inferred. However, the primary goal of the larger study was to assess the feasibility of implementation and demand for the program rather than determining efficacy. Fifth, program participants self-selected into the program, which may have biased findings either upwards or downwards depending on motivation and previously adopted health behaviors. Relatedly, the program’s first-come first-serve enrollment process introduced another important limitation. While over 850 individuals added their names to a waitlist, those highest on the list were the ones who enrolled during the pilot phase of the program due to capacity limits. These patients did not fully mirror the patient population of this safety-net setting, given their high average levels of education, English proficiency, commercial insurance status, and food security, potentially influencing reported behavior change outcomes and limiting the generalizability of findings to the safety-net setting population. It would be important to replicate this study with participants who are more representative of that population. Notwithstanding these limitations, the consistent pattern of positive findings demonstrates the potential of the PBLM program in a safety-net setting. 

## 5. Conclusions

Lifestyle medicine is a proven approach for improving behaviors that drive cardiometabolic health, yet it is underutilized within safety-net healthcare settings that serve patients most at risk for chronic illness. The pilot PBLM program shows promise in improving patient-reported outcomes, including lifestyle behaviors such as healthful plant-based eating, physical activity, stress management, and sleep, within a safety-net healthcare setting. The findings from this study provide important insights for healthcare practitioners implementing similar lifestyle medicine interventions in healthcare settings, including the benefits of a multidisciplinary team, addressing all pillars of lifestyle medicine, and the ability for patients to improve their knowledge, barriers, skills, and behaviors when given adequate support. Additional studies should employ more rigorous methodologies including comparison/control groups and longer follow-up periods to further demonstrate the effectiveness of lifestyle medicine interventions. Declines in behavior adherence over time are common in lifestyle interventions [45]; therefore, studies with longer follow-up periods, particularly after participants have “graduated” from programs, are needed to determine the lasting impact of comprehensive lifestyle medicine interventions. 

## Figures and Tables

**Table 1:** Demographic Characteristics of the PBLM Program Sample (*n* = 71).

	N	Percent or Mean (SD)
**Gender**	71	
Male		32.4
Female		67.6
**Age**	70	55.0 (11.0)
**Race and ethnicity**	70	
American Indian or Alaska Native		1.4
Asian		4.3
Black or African American		31.4
Hispanic or Latino		17.1
Native Hawaiian or Other Pacific Islander		0.0
White		38.6
Two or more		4.3
Other		2.9
**Language use**	71	
English-only		85.9
Spanish-only		7.0
English and Spanish		4.2
Other language		2.8
**Education**	71	
Less than high school		2.8
High school		5.6
Some college		23.9
College/More than college		67.6
**Health insurance**	70	
Private (employer, someone else’s employer, purchased)		57.1
Public (Medicaid, Medicare)		20.0
No insurance		1.4
Other (other, two or more, military, COBRA)		21.4
**Marital status**	71	
Single		43.7
Married/living with a partner		40.8
Separated/divorced/widowed		15.5
**Number of household residents**	71	2.3 (1.2)
**Food security**	71	
Often we don’t have enough to eat		0.0
Sometimes we don’t have enough to eat		1.4
We have enough to eat but not always the kinds of food we want		40.8
We always have enough to eat and the kinds of food we want		57.7

Notes: Numbers of responses may vary due to missing data.

**Table 2:** Nutrition Knowledge, Barriers to Plant-Based Diet, Food and Beverage Consumption, Lifestyle Behaviors, Self-Reported Heath, and Quality-of-Life in PBLM Program Sample (*n* = 71).

	Baseline	3-Month Follow-Up	6-Month Follow-Up	Baseline to 3-Month Change	3- to 6-Month Change	Baseline to 6-Month Change
	Mean (SD) or Percent	Mean (SD) or Percent	Mean (SD) or Percent	*p*-Value	*p*-Value	*p*-Value
**Nutrition Knowledge (% correct)**	66.4	71.5	73.6	*p* = 0.005	*p* = 0.176	*p* = 0.001
**Barriers to Adopting a Plant-Based** **Diet (0–21)**	7.2 (3.9)	5.7 (4.3)	4.8 (3.7)	*p* = 0.002	*p* = 0.005	*p* < 0.001
**Food and Beverage Consumption per Week**						
*Healthier Plant-Based Foods*						
Whole grain foods	5.5 (5.8)	8.3 (6.2)	7.1 (5.9)	*p* < 0.001	*p* = 0.061	*p* = 0.053
Fruits	7.7 (5.9)	11.6 (6.4)	10.2 (6.6)	*p* < 0.001	*p* = 0.076	*p* = 0.002
Vegetables	9.3 (6.3)	12.6 (6.3)	11.2 (6.2)	*p* < 0.001	*p* = 0.044	*p* = 0.008
Nuts or seeds	4.5 (4.9)	4.7 (4.3)	4.7 (4.1)	*p* = 0.690	*p* = 0.934	*p* = 0.754
Legumes, tofu, and tempeh ^1^	2.7 (2.6)	3.8 (2.7)	3.9 (2.6)	*p* < 0.001	*p* = 0.827	*p* < 0.001
*Less Healthy Plant-Based Foods*						
Refined grain foods	3.8 (4.2)	1.9 (2.3)	2.0 (1.7)	*p* < 0.001	*p* = 0.610	*p* < 0.001
Sugar-sweetened beverages ^2^	0.8 (1.5)	0.6 (1.5)	0.4 (0.8)	*p* = 0.183	*p* = 0.316	*p* = 0.011
Sweets	4.0 (4.4)	2.6 (2.8)	2.3 (2.4)	*p* = 0.009	*p* = 0.495	*p* = 0.002
*Animal Foods*						
Dairy	4.2 (5.2)	1.5 (3.3)	1.6 (2.2)	*p* < 0.001	*p* = 0.871	*p* < 0.001
Eggs	2.4 (3.5)	1.0 (1.6)	0.8 (1.3)	*p* < 0.001	*p* < 0.405	*p* < 0.001
Fish/seafood	1.7 (2.3)	1.4 (1.9)	1.0 (1.6)	*p* = 0.186	*p* = 0.072	*p* = 0.021
Meat ^3^	4.7 (5.0)	1.9 (3.3)	2.0 (2.9)	*p* < 0.001	*p* = 0.603	*p* < 0.001
Modified hPDI ^4^	37.8 (7.5)	43.9 (6.3)	43.1 (5.6)	*p* < 0.001	*p* = 0.125	*p* < 0.001
**Lifestyle Behaviors**						
30 min or more of exercise (days)	2.3 (2.1)	2.9 (2.3)	3.0 (2.4)	*p* = 0.017	*p* = 0.824	*p* = 0.036
Media consumption (hours)	3.7 (2.4)	3.2 (1.9)	3.0 (1.6)	*p* = 0.069	*p* = 0.519	*p* = 0.022
Average hours of sleep on a weeknight	6.3 (1.3)	6.4 (1.5)	6.5 (1.3)	*p* = 0.373	*p* = 0.494	*p* = 0.058
Average hours of sleep on a weekend night	7.3 (1.9)	6.9 (1.6)	7.0 (1.5)	*p* = 0.084	*p* = 0.764	*p* = 0.134
Sleep quality in the last month (fairly good/very good)	54.9	64.8	67.1	*p* = 0.118	*p* = 0.774	*p* = 0.035
**Self-rated health (0–10)**	5.9 (1.9)	6.6 (1.8)	6.5 (2.2)	*p* = 0.001	*p* = 0.447	*p* = 0.038
**Quality of life (good/very good)**	53.5	65.7	66.2	*p* = 0.108	*p* = 1.00	*p* = 0.108

Notes: ^1^ Legumes, tofu, and tempeh = number of times legumes, tofu, and tempeh were consumed per week during previous 7 days. ^2^ Sugar-sweetened beverages = number of times regular soda or punch/energy drinks/sweetened fruit drinks were consumed per week during the previous 7 days. ^3^ Meat = number of times red meat/pork, processed meat, and chicken were consumed per week during the previous 7 days. ^4^ Modified hPDI = total score of healthier plant-based foods, less healthy plant-based foods, and animal foods.

## Data Availability

The raw data supporting the conclusions of this article will be made available by the authors, without undue reservation.

## Supplementary Materials

The following supporting information can be downloaded at: https://www.mdpi.com/article/10.3390/nu15132857/s1. Table S1: Barriers to Adopting a Plant-Based Diet (*n* = 71).

## References

[1] GBD 2017 Diet Collaborators (2019). Health effects of dietary risks in 195 countries, 1990–2017: A systematic analysis for the Global Burden of Disease Study 2017. Lancet.

[2] The US Burden of Disease Collaborators (2018). The State of US Health, 1990–2016: Burden of Diseases, Injuries, and Risk Factors Among US States. JAMA.

[3] Katz D.L., Frates E.P., Bonnet J.P., Gupta S.K., Vartiainen E., Carmona R.H. (2018). Lifestyle as Medicine: The Case for a True Health Initiative. Am. J. Health Promot..

[4] Benigas S., Shurney D., Stout R. (2022). Making the Case for Lifestyle Medicine. Suppl. J. Fam. Pract..

[5] Satija A., Bhupathiraju S.N., Spiegelman D., Chiuve S.E., Manson J.E., Willett W., Rexrode K.M., Rimm E.B., Hu F.B. (2017). Healthful and Unhealthful Plant-Based Diets and the Risk of Coronary Heart Disease in U.S. Adults. J. Am. Coll. Cardiol..

[6] Dinu M., Abbate R., Gensini G.F., Casini A., Sofi F. (2017). Vegetarian, vegan diets and multiple health outcomes: A systematic review with meta-analysis of observational studies. Crit. Rev. Food Sci. Nutr..

[7] Melina V., Craig W., Levin S. (2016). Position of the Academy of Nutrition and Dietetics: Vegetarian Diets. J. Acad. Nutr. Diet..

[8] Qian F., Liu G., Hu F.B., Bhupathiraju S.N., Sun Q. (2019). Association Between Plant-Based Dietary Patterns and Risk of Type 2 Diabetes: A Systematic Review and Meta-analysis. JAMA Intern. Med..

[9] Kim H., Caulfield L.E., Garcia-Larsen V., Steffen L.M., Coresh J., Rebholz C.M. (2019). Plant-Based Diets Are Associated With a Lower Risk of Incident Cardiovascular Disease, Cardiovascular Disease Mortality, and All-Cause Mortality in a General Population of Middle-Aged Adults. J. Am. Heart Assoc..

[10] Turner-McGrievy G., Mandes T., Crimarco A. (2017). A plant-based diet for overweight and obesity prevention and treatment. J. Geriatr. Cardiol..

[11] Ornish D., Scherwitz L.W., Billings J.H., Brown S.E., Gould K.L., Merritt T.A., Sparler S., Armstrong W.T., Ports T.A., Kirkeeide R.L. (1998). Intensive lifestyle changes for reversal of coronary heart disease. JAMA.

[12] Aldana S.G., Greenlaw R.L., Diehl H.A., Salberg A., Merrill R.M., Ohmine S., Thomas C. (2006). The behavioral and clinical effects of therapeutic lifestyle change on middle-aged adults. Prev. Chronic Dis..

[13] Hauser M.E., McMacken M., Lim A., Shetty P. (2022). Nutrition-An Evidence-Based, Practical Approach to Chronic Disease Prevention and Treatment. Suppl. J. Fam. Pract..

[14] Kelly J., Karlsen M., Steinke G. (2020). Type 2 Diabetes Remission and Lifestyle Medicine: A Position Statement From the American College of Lifestyle Medicine. Am. J. Lifestyle Med..

[15] ElSayed N.A., Aleppo G., Aroda V.R., Bannuru R.R., Brown F.M., Bruemmer D., Collins B.S., Hilliard M.E., Isaacs D., Johnson E.L. (2023). Facilitating Positive Health Behaviors and Well-being to Improve Health Outcomes: Standards of Care in Diabetes—2023. Diabetes Care.

[16] Blonde L., Umpierrez G.E., Reddy S.S., McGill J.B., Berga S.L., Bush M., Chandrasekaran S., DeFronzo R.A., Einhorn D., Galindo R.J. (2022). American Association of Clinical Endocrinology Clinical Practice Guideline: Developing a Diabetes Mellitus Comprehensive Care Plan—2022 Update. Endocr. Pract..

[17] Arnett D.K., Blumenthal R.S., Albert M.A., Buroker A.B., Goldberger Z.D., Hahn E.J., Himmelfarb C.D., Khera A., Lloyd-Jones D., McEvoy J.W. (2019). 2019 ACC/AHA Guideline on the Primary Prevention of Cardiovascular Disease: A Report of the American College of Cardiology/American Heart Association Task Force on Clinical Practice Guidelines. Circulation.

[18] Lichtenstein A.H., Appel L.J., Vadiveloo M., Hu F.B., Kris-Etherton P.M., Rebholz C.M., Sacks F.M., Thorndike A.N., Horn L.V., Wylie-Rosett J. (2021). 2021 Dietary Guidance to Improve Cardiovascular Health: A Scientific Statement From the American Heart Association. Circulation.

[19] Belardo D., Michos E.D., Blankstein R., Blumenthal R.S., Ferdinand K.C., Hall K., Klatt K., Natajaran P., Ostfeld R.J., Reddy K. (2022). Practical, Evidence-Based Approaches to Nutritional Modifications to Reduce Atherosclerotic Cardiovascular Disease: An American Society For Preventive Cardiology Clinical Practice Statement. Am. J. Prev. Cardiol..

[20] Jacobson T.A., Maki K.C., Orringer C.E., Jones P.H., Kris-Etherton P., Sikand G., La Forge R., Daniels S.R., Wilson D.P., Morris P.B. (2015). National Lipid Association Recommendations for Patient-Centered Management of Dyslipidemia: Part 2. J. Clin. Lipidol..

[21] Rock C.L., Thomson C., Gansler T., Gapstur S.M., McCullough M.L., Patel A.V., Andrews K.S., Bandera E.V., Spees C.K., Robien K. (2020). American Cancer Society guideline for diet and physical activity for cancer prevention. CA Cancer J. Clin..

[22] American College of Lifestyle Medicine Health Systems Council. https://lifestylemedicine.org/ACLM/Partners/Health-Systems-Council/ACLM/Partners/Health-Systems/Health-Systems-Council.aspx?hkey=ca721dcc-8bfa-457c-b1cc-ee10e7866172.

[23] Frieden T.R., Centers for Disease Control and Prevention (2013). CDC Health Disparities and Inequalities Report—United States, 2013. Foreword. MMWR Suppl..

[24] Caraballo C., Herrin J., Mahajan S., Massey D., Lu Y., Ndumele C.D., Drye E.E., Krumholz H.M. (2022). Temporal Trends in Racial and Ethnic Disparities in Multimorbidity Prevalence in the United States, 1999–2018. Am. J. Med..

[25] Ein L.M., Altman S. (2000). Institute of Medicine Committee on the Changing Market Managed Care and the Future Viability of Safety Net Providers. Americas’s Health Care Safety Net: Intact but Endangered.

[26] Health Resources & Services Administration Health Center Program: Impact and Growth. https://bphc.hrsa.gov/about-health-centers/health-center-program-impact-growth.

[27] Nguyen J.Y., Major J.M., Knott C.J., Freeman K.M., Downs T.M., Saxe G.A. (2006). Adoption of a plant-based diet by patients with recurrent prostate cancer. Integr. Cancer Ther..

[28] Gill D.P., Blunt W., Boa Sorte Silva N.C., Stiller-Moldovan C., Zou G.Y., Petrella R.J. (2019). The HealtheSteps™ lifestyle prescription program to improve physical activity and modifiable risk factors for chronic disease: A pragmatic randomized controlled trial. BMC Public Health.

[29] Knutsen S.F., Knutsen R. (1991). The Tromso Survey: The Family Intervention study--the effect of intervention on some coronary risk factors and dietary habits, a 6-year follow-up. Prev. Med..

[30] Cupples M.E., McKnight A. (1994). Randomised controlled trial of health promotion in general practice for patients at high cardiovascular risk. BMJ.

[31] Gallagher R., Kirkness A., Zelestis E., Hollams D., Kneale C., Armari E., Bennett T., Daly J., Tofler G. (2012). A randomised trial of a weight loss intervention for overweight and obese people diagnosed with coronary heart disease and/or type 2 diabetes. Ann. Behav. Med..

[32] Katcher H.I., Ferdowsian H.R., Hoover V.J., Cohen J.L., Barnard N.D. (2010). A worksite vegan nutrition program is well-accepted and improves health-related quality of life and work productivity. Ann. Nutr. Metab..

[33] Elkoustaf R.A., Aldaas O.M., Batiste C.D., Mercer A., Robinson M., Newton D., Burchett R., Cornelius C., Patterson H., Ismail M.H. (2019). Lifestyle Interventions and Carotid Plaque Burden: A Comparative Analysis of Two Lifestyle Intervention Programs in Patients with Coronary Artery Disease. Perm. J..

[34] Carroll S., Borkoles E., Polman R. (2007). Short-term effects of a non-dieting lifestyle intervention program on weight management, fitness, metabolic risk, and psychological well-being in obese premenopausal females with the metabolic syndrome. Appl. Physiol. Nutr. Metab..

[35] Kaplan R.M., Hartwell S.L., Wilson D.K., Wallace J.P. (1987). Effects of diet and exercise interventions on control and quality of life in non-insulin-dependent diabetes mellitus. J. Gen. Intern. Med..

[36] Albert S.L., Massar R.E., Kwok L., Correa L., Polito-Moller K., Joshi S., Shah S., McMacken M. (2022). Pilot Plant-Based Lifestyle Medicine Program in an Urban Public Healthcare System: Evaluating Demand and Implementation. Am. J. Lifestyle Med..

[37] Albert S.L., Massar R.E., Correa L., Kwok L., Joshi S., Shah S., Boas R., Alcalá H.E., McMacken M. (2023). Change in cardiometabolic risk factors in a pilot safety-net plant-based lifestyle medicine program. Front. Nutr..

[38] Lea E.J., Crawford D., Worsley A. (2006). Public views of the benefits and barriers to the consumption of a plant-based diet. Eur. J. Clin. Nutr..

[39] Rifas-Shiman S.L., Willett W.C., Lobb R., Kotch J., Dart C., Gillman M.W. (2001). PrimeScreen, a brief dietary screening tool: Reproducibility and comparability with both a longer food frequency questionnaire and biomarkers. Public. Health Nutr..

[40] American College of Lifestyle Medicine, Loma Linda University Health (2017). Lifestyle Assessment Long Form Physician Version. https://ihacares.com/assets/pdfs/Lifestyle%20Medicine/ACLM%20LLU%20Long%20Form.pdf.

[41] Center for Disease Control and Prevention (2017). Youth Risk Behavior Survey Questionnaire.

[42] Buysse D.J., Reynolds C.F., Monk T.H., Berman S.R., Kupfer D.J. (1989). The Pittsburgh Sleep Quality Index: A new instrument for psychiatric practice and research. Psychiatry Res..

[43] The WHOQOL Group (1998). Development of the World Health Organization WHOQOL-BREF quality of life assessment. Psychol. Med..

[44] StataCorp (2015). Stata Statistical Software: Release 14.

[45] Middleton K.R., Anton S.D., Perri M.G. (2013). Long-Term Adherence to Health Behavior Change. Am. J. Lifestyle Med..

[46] Massar S.A.A., Liu J.C.J., Mohammad N.B., Chee M.W.L. (2017). Poor habitual sleep efficiency is associated with increased cardiovascular and cortisol stress reactivity in men. Psychoneuroendocrinology.

[47] Fine L.J., Philogene G.S., Gramling R., Coups E.J., Sinha S. (2004). Prevalence of multiple chronic disease risk factors. 2001 National Health Interview Survey. Am. J. Prev. Med..

[48] Hargens T.A., Kaleth A.S., Edwards E.S., Butner K.L. (2013). Association between sleep disorders, obesity, and exercise: A review. Nat. Sci. Sleep..

[49] Watson N.F. (2015). Sleep duration: A consensus conference. J. Clin. Sleep. Med..

[50] Dedhia P., Maurer R. (2022). Sleep and Health-A Lifestyle Medicine Approach. J. Fam. Pract..

[51] Tylka T.L., Annunziato R.A., Burgard D., Daníelsdóttir S., Shuman E., Davis C., Calogero R.M. (2014). The weight-inclusive versus weight-normative approach to health: Evaluating the evidence for prioritizing well-being over weight loss. J. Obes..

[52] Alberga A.S., Edache I.Y., Forhan M., Russell-Mayhew S. (2019). Weight bias and health care utilization: A scoping review. Prim. Health Care Res. Dev..

[53] Dolor R.J., Østbye T., Lyna P., Coffman C.J., Alexander S.C., Tulsky J.A., Brouwer R.J., Esoimeme I., Pollak K.I. (2010). What are physicians’ and patients’ beliefs about diet, weight, exercise, and smoking cessation counseling?. Prev. Med..

[54] Morton K.F., Pantalos D.C., Ziegler C., Patel P.D. (2022). Whole-Foods, Plant-Based Diet Perceptions of Medical Trainees Compared to Their Patients: A Cross-Sectional Pilot Study. Am. J. Lifestyle Med..

[55] Kirkpatrick S.I., Reedy J., Butler E.N., Dodd K.W., Subar A.F., Thompson F.E., McKinnon R.A. (2014). Dietary assessment in food environment research: A systematic review. Am. J. Prev. Med..

