# Supplementary material for: Patient-Reported Outcomes from a Pilot Plant-Based Lifestyle Medicine Program in a Safety-Net Setting
Source: Nutrients. 2023 Jun 24;15(13):2857. doi: 10.3390/nu15132857 (PMC10343841; doi:10.3390/nu15132857)
Supplement: Supplementary file 1 [file nutrients-15-02857-s001.zip › nutrients-2408119-supplementary.pdf]

Table S1. Barriers to Adopting a Plant-Based Diet (n=71)

	**Baseline**		**3-Month Follow-Up**		**6-Month Follow-Up**		**Baseline to 3-Month Change**	**3- to 6-Month Change**	**Baseline to 6-Month Change**
**Agree/Strongly agree**	**N**	**Percent**	**N**	**Percent**	**N**	**Percent**	**p-value**	**p-value**	**p-value**
You are not sure how to be healthy on a plant-based diet	68	52.9	68	29.4	69	13.0	p < 0.001	p = 0.003	p < 0.001
You don't want to change your eating habits or routine	71	7.0	71	7.0	70	7.1	p = 1.00	p = 1.00	p = 1.00
Your family or partner won't eat a plant-based diet	58	51.7	58	43.1	57	40.4	p = 0.332	p = 1.00	p = 0.503
There is not enough choice when you eat out	70	68.6	70	67.1	70	60.0	p = 1.00	p = 0.227	p = 0.189
You do not have enough willpower to change to a plant-based diet	69	26.1	69	20.3	70	12.9	p = 0.454	p = 0.227	p = 0.031
Someone else decides on most of the food you eat	71	11.3	71	4.2	71	8.5	p = 0.125	p = 0.453	p = 0.727
It would be too expensive	68	30.9	68	30.9	69	23.2	p = 1.00	p = 0.180	p = 0.227
You don't like the taste of many of the foods that are the foundation of a plant-based diets	69	8.7	69	10.1	70	7.1	p = 1.00	p = 0.500	p = 1.00
You would have to go food shopping too often	70	42.9	70	45.7	70	40.0	p = 0.815	p = 0.267	p = 1.00
You would get indigestion, bloating, or gas	65	24.6	65	24.6	69	27.5	p = 1.00	p = 1.00	p = 1.00
It would not be filling enough	69	17.4	69	14.5	69	13.0	p = 0.791	p = 1.00	p = 1.00
You would (or do) crave meat, dairy, or eggs	71	46.5	71	39.4	71	31.0	p = 0.302	p = 0.210	p = 0.013
You don't know how to prepare plant-based meals	69	55.1	69	29.0	68	20.6	p < 0.001	p = 0.180	p < 0.001
It would not be tasty enough	67	17.9	67	16.4	69	15.9	p = 1.00	p = 1.00	p = 0.791
The plant-based foods you would need aren't available where you shop	69	27.5	69	23.2	71	28.2	p = 0.549	p = 0.774	p = 1.00
You don't know what to eat on a plant-based diet	69	53.6	69	17.4	69	11.6	p < 0.001	p = 0.219	p < 0.001
It takes too long to prepare plant-based meals	62	30.6	62	30.6	70	30.0	p = 1.00	p = 1.00	p = 1.00
It takes too much planning to eat a plant-based diet	63	54.0	63	49.2	70	42.9	p = 0.607	p = 0.607	p = 0.092
You don't know anyone who eats a plant-based diet	71	43.7	71	36.6	71	28.2	p = 0.405	p = 0.210	p = 0.035
You haven't read or heard much about a plant-based diet	71	25.4	71	9.9	70	2.9	p < 0.001	p = 0.125	p < 0.001
Your friends or family encourage you to eat unhealthy foods with them	70	41.4	70	35.7	69	31.9	p = 0.556	p = 0.629	p = 0.210

NOTES: Number of responses may vary due to missing data.
